# Combined therapeutic strategy of balloon pulmonary angioplasty before and after pulmonary endarterectomy in a patient with severe chronic thromboembolic pulmonary hypertension with Kartagener’s syndrome: a case report

**DOI:** 10.1093/ehjcr/ytaf349

**Published:** 2025-07-25

**Authors:** Tomohiro Kasahara, Jun Yamashita, Ikki Komatsu, Yusuke Shimahara, Kazuhiro Satomi

**Affiliations:** Department of Cardiology, Tokyo Medical University, 6-7-1 Nishi-shinjuku, Shinjuku-ku, Tokyo 160-0023, Japan; Department of Cardiology, Tokyo Medical University, 6-7-1 Nishi-shinjuku, Shinjuku-ku, Tokyo 160-0023, Japan; Department of Cardiology, Tokyo Medical University, 6-7-1 Nishi-shinjuku, Shinjuku-ku, Tokyo 160-0023, Japan; Department of Cardiovascular Surgery, Tokyo Medical University, 6-7-1 Nishi-shinjuku, Shinjuku-ku, Tokyo 160-0023, Japan; Department of Cardiology, Tokyo Medical University, 6-7-1 Nishi-shinjuku, Shinjuku-ku, Tokyo 160-0023, Japan

**Keywords:** Chronic thromboembolic pulmonary hypertension, Kartagener’s syndrome, Balloon pulmonary angioplasty, Pulmonary endarterectomy, Combined treatment, Case report

## Abstract

**Background:**

This case report describes the novel use of balloon pulmonary angioplasty before and after pulmonary endarterectomy for treating severe chronic thromboembolic pulmonary hypertension in a patient with Kartagener's syndrome. Chronic thromboembolic pulmonary hypertension is recognized as one of the few treatable types of pulmonary hypertension, and pulmonary endarterectomy is the standard treatment. However, advanced age (e.g. >75 years) and certain comorbidities may make it difficult to perform pulmonary endarterectomy, and balloon pulmonary angioplasty and medical therapy can be alternative options in such cases.

**Case summary:**

A 63-year-old woman with Kartagener’s syndrome was diagnosed with severe chronic thromboembolic pulmonary hypertension after presenting with dyspnoea on exertion. She underwent preceding balloon pulmonary angioplasty without any complications to reduce the perioperative risk of pulmonary endarterectomy. An additional balloon pulmonary angioplasty after pulmonary endarterectomy was performed for residual dyspnoea, which led to improvement of the overall condition.

**Discussion:**

Balloon pulmonary angioplasty before pulmonary endarterectomy may be indicated for high-risk cases for perioperative risk reduction. This report highlights the usefulness of the combination of balloon pulmonary angioplasty before and after pulmonary endarterectomy for the successful treatment of severe chronic thromboembolic pulmonary hypertension in a patient with Kartagener’s syndrome, which may have various perioperative risks.

Learning points

- Balloon pulmonary angioplasty before pulmonary endarterectomy can reduce perioperative risks for severe chronic thromboembolic pulmonary hypertension.
- Combining balloon pulmonary angioplasty before and after pulmonary endarterectomy may enhance outcomes in patients with residual pulmonary hypertension or dyspnoea.

## Introduction

Chronic thromboembolic pulmonary hypertension (CTEPH) is a rare but critical disease characterized by narrowing and occlusion of the pulmonary arteries (PAs) by organized thrombi.^1^ Surgical pulmonary endarterectomy (PEA) is the standard therapy for CTEPH,^2,3^ but severe pulmonary hypertension (PH) with right heart deterioration can increase the risk of adverse perioperative events.^4^ In selected situations, balloon pulmonary angioplasty (BPA) is attempted before PEA to decrease PH preoperatively.

Kartagener’s syndrome (KS) is a rare clinical condition. Pulmonary endarterectomy may be challenging in KS because of confusion and technical difficulties associated with reversed right–left laterality. In KS, the combination of situs inversus, bronchiectasis, and chronic sinusitis can markedly increase the technical difficulty and perioperative risks associated with invasive procedures such as PEA.

Herein, we report a case of severe CTEPH complicated by KS that was successfully treated with combined therapy of BPA before and after PEA.

## Summary figure

BPA, balloon pulmonary angioplasty; CO, cardiac output; CT, computed tomography; CTEPH, chronic thromboembolic pulmonary hypertension; KS, Kartagener’s syndrome; MPAP, mean pulmonary artery pressure; M, months; PEA, pulmonary endarterectomy; PVR, pulmonary vascular resistance; RHC, right heart catheterization.

**Figure:**
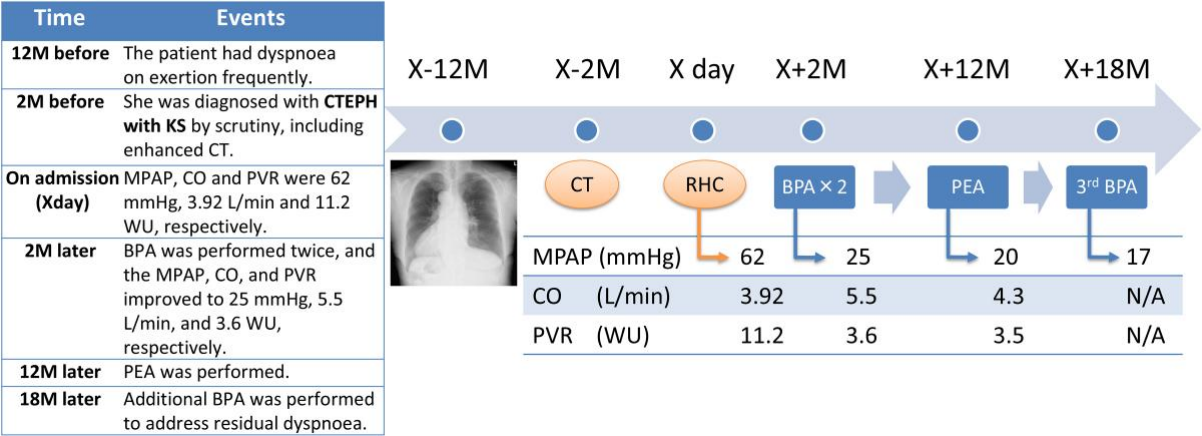


## Case presentation

**Figure 1:**
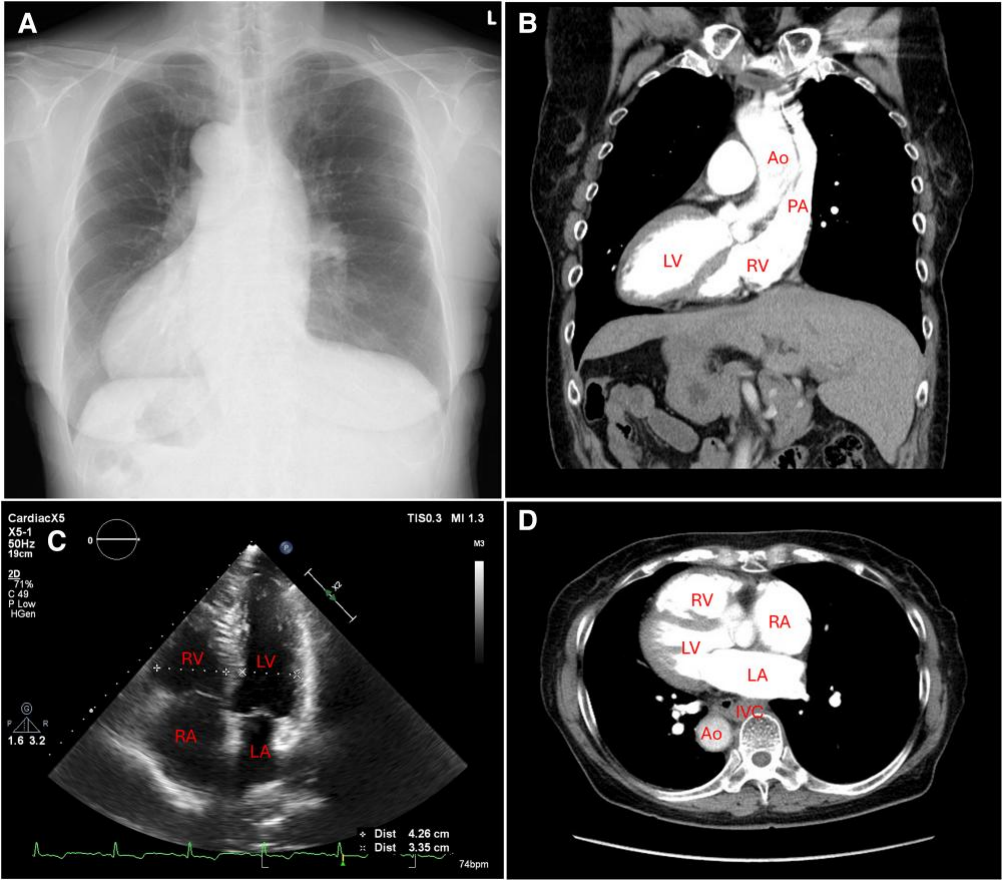
Complete situs inversus on chest X-ray, thoracic echocardiography, and computed tomography. (*A*) Pre-treatment chest X-ray, frontal view. (*B*) Pre-treatment chest computed tomography, coronal view. (*C*) Pre-treatment *trans*-thoracic echocardiogram, four-chamber view. (*D*) Pre-treatment chest computed tomography, axial view. Ao, aorta; IVC, inferior vena cava; LA, left atrium; LV, left ventricle; PA, pulmonary artery; RA, right atrium; RV, right ventricle.

A 63-year-old woman presented to a hospital with a 2-year history of severe dyspnoea on exertion. She had been diagnosed with KS with the triad of chronic sinusitis, bronchiectasis, and complete situs inversus (CSI) with abnormal venous reflux (*Figure 1A*). Complete situs inversus made dextrocardia, which was mirror image positioning of the heart (*Figure 1B–D*). Pulmonary embolism was diagnosed using enhanced computed tomography (CT) and pulmonary perfusion scintigraphy, which revealed thrombi in the bilateral PAs and multiple segmental blood-flow defects. Anticoagulation with edoxaban for 3 months failed to improve symptoms, and follow-up CT showed no remarkable changes. She was diagnosed with CTEPH without thrombotic predisposition and referred to our institution for further evaluation and treatment.

**Figure 2:**
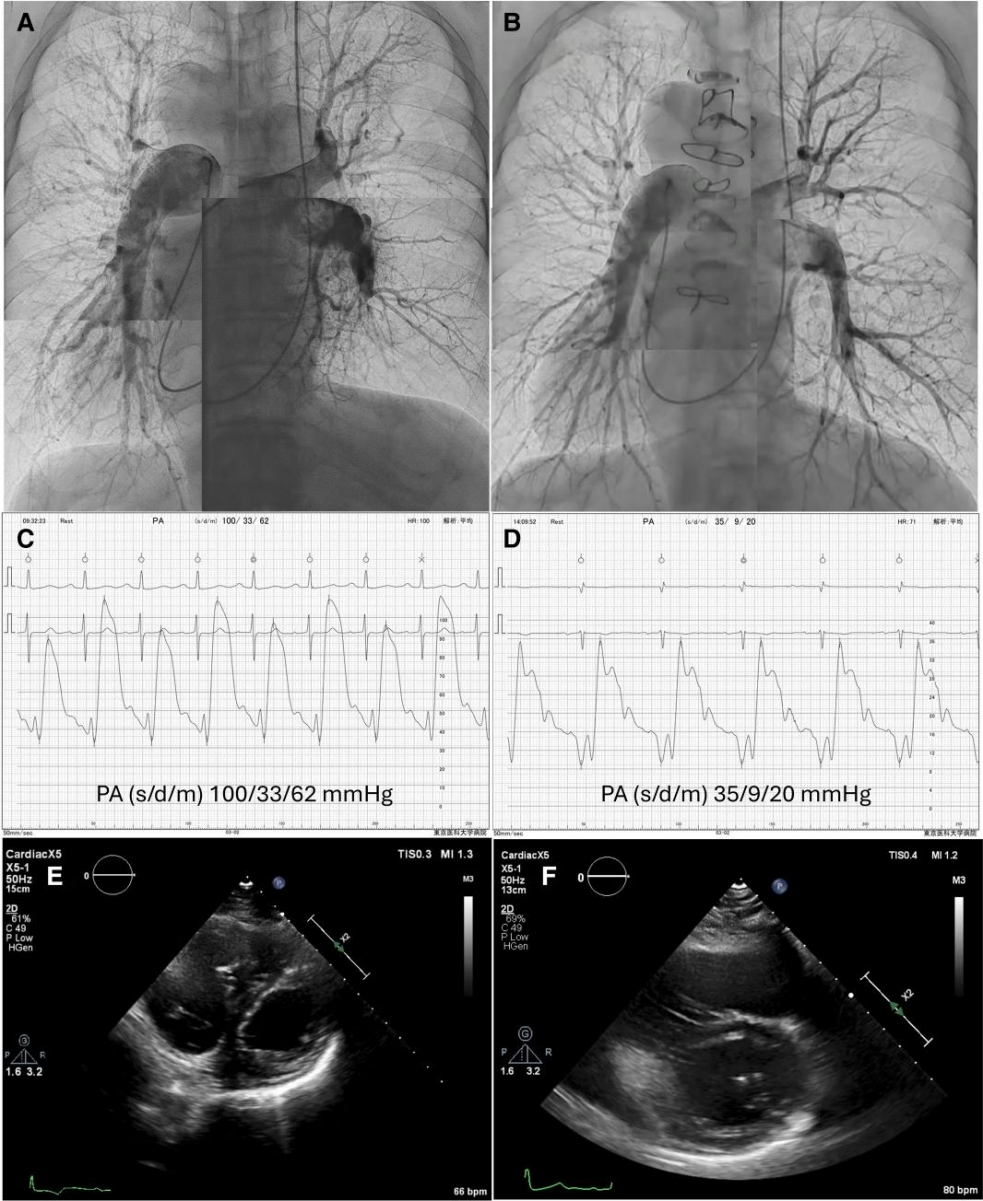
Pulmonary angiography, curve of pulmonary arterial pressure, and thoracic echocardiography before and after two balloon angioplasties and pulmonary endarterectomy. (*A*) Initial pulmonary angiography. (*B*) Pulmonary angiography after two balloon angioplasties and pulmonary endarterectomy. (*C*) Initial curve of pulmonary arterial pressure. (*D*) Curve of pulmonary arterial pressure after two balloon angioplasties and pulmonary endarterectomy. (*E*) Initial short-axis view in thoracic echocardiography when the patient lay down on right side. (*F*) Short-axis view in thoracic echocardiography after two balloon angioplasties and pulmonary endarterectomy. PA, pulmonary artery.

On admission, the oxygen saturation was 97% with 3 L of oxygen, and she could walk 185 m in the 6-min walking test. Transthoracic echocardiography showed a D-shaped left ventricle with preserved ejection fraction, enlarged impaired right ventricle with 14 mm of TAPSE and 7.0 cm/s of systolic wave prime, and tricuspid regurgitation with a pressure gradient of 74 mmHg. Mean PA pressure (MPAP), cardiac output (CO), and pulmonary vessel resistance (PVR) were 62 mmHg, 3.92 L/min, and 11.2 Wood units (WU), respectively. Pulmonary angiography revealed distal and proximal lesions in the bilateral PAs, respectively (*Figure 2A, C*, and *E*). As the risk of adverse perioperative events was thought to be high because of high MPAP and PVR with KS and CSI, our CTEPH team comprising cardiovascular surgeons and cardiologists decided to perform BPA for perioperative risk reduction before PEA.

**Figure 3:**
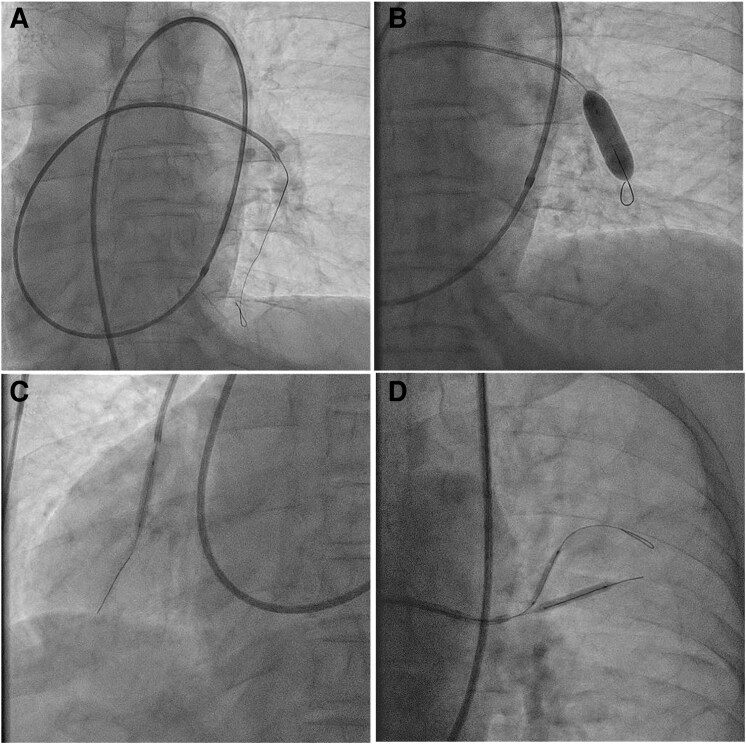
Access and lesions dilated by balloon in balloon pulmonary angioplasty.

The BPA procedures were conducted as previously reported.^5^ During the first BPA procedure, access was obtained through the left femoral vein (FV). The catheter was advanced from the left FV through the superior vena cava, followed by the left PA, creating a loop in the right atrium/right ventricle supported by an extension catheter (*Figure 3A*). The web of A4, web- and ring-like stenosis of A5, and subtotal lesion of A9 was sequentially dilated, gradually increasing in size from 1.5 to 5 mm. The severe stenotic lesion in the lobar artery, also a target site for PEA, was dilated using an 8 mm balloon (*Figure 3B*). For the second BPA, access was obtained through the left jugular vein (JV). Web- and ring-like stenoses in A1, A2, A9, and A10 in the right PA and ones A1, A2, and A3 in the left PA were dilated in size from 2 to 4 mm (*Figure 3C* and *D*). Procedures were completed without complications (*Table 1*). Subsequently, MPAP, CO, and PVR improved to 25 mmHg, 5.5 L/min, and 3.6 WU, respectively. Riociguat 1.5 mg/day was added after the second BPA.

**Table 1:** Treatment segments of balloon pulmonary angioplasty and mean pulmonary artery pressure of pre- and post-balloon pulmonary angioplasty

	Target arteries	1st BPA	2nd BPA	3rd BPA
Right	A1		〇(3, 4 mm)	〇(4 mm)
	A2		〇(3, 4 mm)	〇(3 mm)
	A3			
	A4			
	A5			
	A6			
	(A7)	-	-	-
	A8			〇(4 mm)
	A9		〇(2, 3 mm)	〇(2, 3, 4 mm)
	A10		〇(3 mm)	〇(2, 4 mm)
Left	A1		〇(2, 3, 4 mm)	
	A2		〇(3 mm)	
	A3		〇(2, 3 mm)	
	A4	〇(3 mm)		
	A5	〇(3, 5 mm)		
	A6			
	A7			
	A8			
	A9	〇(1.5, 3, 4, 5, 8 mm)		
	A10			
MPAP (mmHg)	Pre-BPA	62	34	17
	Post-BPA	32	25	17

Treatment arteries are marked with ○, and the diameters of the balloons used are indicated in parentheses. In this case, the right A7 corresponds to the usual left A7, and the corresponding pulmonary artery is absent, as indicated by ‘-’.

BPA, balloon pulmonary angioplasty; MPAP, mean pulmonary arterial pressure.

**Figure 4:**
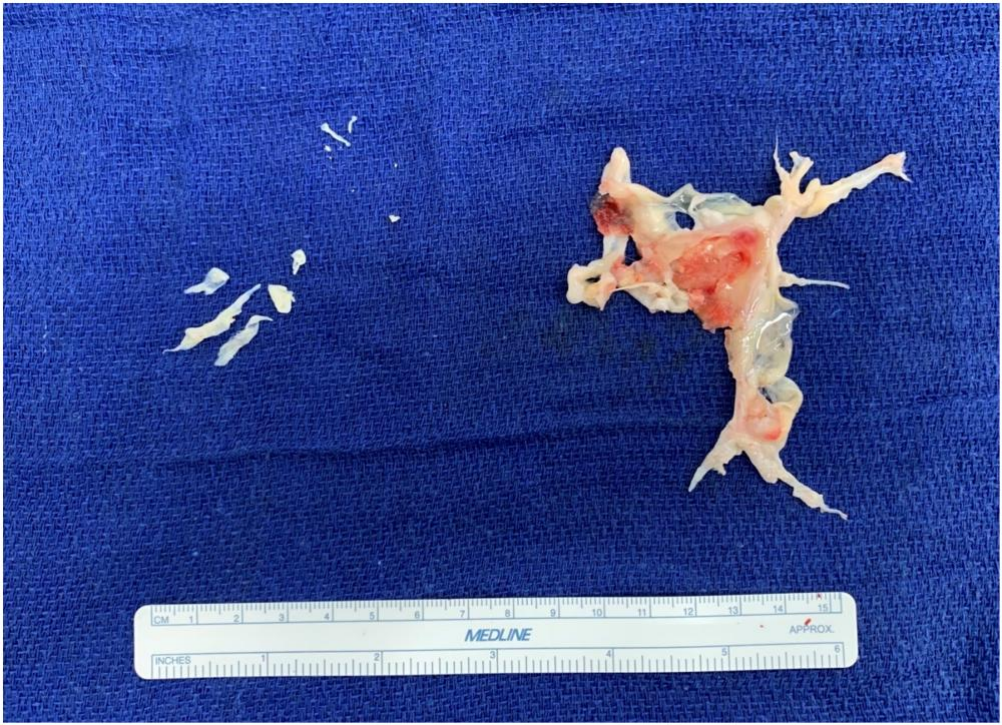
Thrombi and endomembrane obtained by pulmonary endarterectomy. Level 3 on the right. Levels 1 and 2 on the left.^6^

Pulmonary endarterectomy was performed ∼10 months later (*Figure 4*) due to COVID-19 infection. No adverse effects of the BPAs were observed. Anticoagulation was changed to warfarin, and riociguat was terminated; MPAP, CO, and PVR were 20 mmHg, 4.3 L/min, and 3.5 WU, respectively, after PEA (*Figure 2B, D*, and *F*). As effort dyspnoea and desaturation persisted, a third BPA was performed for residual lesions in left PAs from the left JV (*Table 1*). Final MPAP was 17 mm Hg, and her symptoms and desaturation completely disappeared. She receives edoxaban and riociguat 1.5 mg/day and attends a pulmonary vascular outpatient clinic.

## Discussion

To the best of our knowledge, this is the first case report describing BPA and PEA for the treatment of CTEPH complicated by KS. Our treatment strategy of BPA before and after PEA was successful for severe CTEPH with a challenging anatomy and ciliary dyskinesia associated with high-risk of respiratory infection. Pulmonary endarterectomy is the standard treatment for CTEPH. However, patients with advanced age (e.g. >75 years) or high operative risk with poor systemic conditions or comorbidities may hesitate to undergo PEA or choose to undergo only BPA or medical therapy. Balloon pulmonary angioplasty and medical therapy are established treatments for inoperable CTEPH,^7,8^ but the efficacy of BPA before PEA has not been established.^9^ Pulmonary endarterectomy in patients with a poor systemic condition and haemodynamic indices is associated with an increased risk of perioperative death and complications.^1^ In this case, the perioperative risk of PEA was high due to the absence of a history of acute pulmonary embolism or deep venous thrombosis, high MPAP, and complications of KS due to ciliary dyskinesia and CSI. Ciliary dyskinesia affects the ability to excrete sputum, and bronchiectasis complications are a risk factor for PEA. Complete situs inversus does not increase the risk of complications of cardiac surgery, including coronary artery bypass grafting (CABG)^10^; however, KS is a risk factor for respiratory complications in those with congenital heart disease.^11,12^ Pulmonary endarterectomy is not performed in many patients and its association with a high perioperative risk in the present case (even if CSI was not a risk factor for CABG) could not be ruled out. The local CTEPH team addressed these concerns and performed BPA followed by PEA to minimize the operative risk of PEA.

Balloon pulmonary angioplasty can treat peripheral lesions that cannot be reached by PEA, but the risk of complications may be higher in patients with poor haemodynamic status such as high MPAP. The success of PEA, regardless of the site of BPA, was reviewed among 21 patients who underwent PEA after BPA; only a few perioperative complications were reported. However, this was a single-centre, retrospective, observational study, and there are limited reports on treatment strategies such as BPA before PEA. Kirkby *et al*.^13^ reported the effectiveness of BPA before PEA was inferior to BPA for inoperative CTEPH. Consequently, the optimal sites for BPA treatment before PEA in high-risk patients, the frequency of BPA procedures, and the ideal interval between subsequent BPA and PEA have not been established.^4^

In this case, there were some difficulties regarding the approach site due to abnormal venous return, but we could perform BPA as usual using both the FV and JV approaches. However, there may be catheter backup problems, and the JV approach may be superior depending on the treatment site. Therefore, it is important to confirm the anatomy with pre-operative CT.

Regarding BPA after PEA, BPA for residual PH after PEA can improve the haemodynamic status and subjective symptoms.^5^ It was also effective in improving the symptoms in this case without any adverse effects.

## Lead author biography


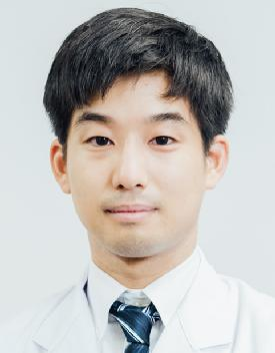
Dr Tomohiro Kasahara is interventional cardiologist at Tokyo Medical University in Japan. His main areas of interest include percutaneous coronary intervention and structural heart disease intervention.

## Data Availability

Due to patient confidentiality and ethical restrictions, the data related to this case report are not publicly available. Anonymized data can be provided by the corresponding author upon reasonable request and with appropriate ethical approvals.
